# Filament Type Recognition for Additive Manufacturing Using a Spectroscopy Sensor and Machine Learning

**DOI:** 10.3390/s25051543

**Published:** 2025-03-02

**Authors:** Gorkem Anil Al, Uriel Martinez-Hernandez

**Affiliations:** 1 Department of Electronic and Electrical Engineering, University of Bath, Bath BA2 7AY, UK; g.al@bath.ac.uk; 2 Multimodal Interaction and Robot Active Perception (Inte-R-Action) Lab, University of Bath, Bath BA2 7AY, UK

**Keywords:** filament recognition, spectroscopy sensor, machine learning, autonomous additive manufacturing

## Abstract

This study presents a novel approach for filament recognition in fused filament fabrication (FFF) processes using a multi-spectral spectroscopy sensor module combined with machine learning techniques. The sensor module measures 18 wavelengths spanning the visible to near-infrared spectra, with a custom-designed shroud to ensure systematic data collection. Filament samples include polylactic acid (PLA), thermoplastic polyurethane (TPU), thermoplastic copolyester (TPC), carbon fibre, acrylonitrile butadiene styrene (ABS), and ABS blended with Carbon fibre. Data are collected using the Triad Spectroscopy module AS7265x (composed of AS72651, AS72652, AS72653 sensor units) positioned at three measurement distances (12 mm, 16 mm, 20 mm) to evaluate recognition performance under varying configurations. Machine learning models, including k-Nearest Neighbors (kNN), Logistic Regression, Support Vector Machine (SVM), and Multi-Layer Perceptron (MLP), are employed with hyperparameter tuning applied to optimise classification accuracy. Results show that the data collected on the AS72651 sensor, paired with the SVM model, achieves the highest accuracy of 98.95% at a 20 mm measurement distance. This work introduces a compact, high-accuracy filament recognition module that can enhance the autonomy of multi-material 3D printing by dynamically identifying and switching between different filaments, optimising printing parameters for each material, and expanding the versatility of additive manufacturing applications.

## 1. Introduction

Additive manufacturing (AM), known as 3D printing, has revolutionised industries by enabling rapid prototyping and custom production through a precise layer-by-layer process. This technique builds objects directly from digital models by depositing materials, such as plastics, metals, or composites, one layer at a time [1,2,3]. Among various AM techniques, fused filament fabrication (FFF) is one of the most widely used methods, particularly due to its ability to produce prototypes and functional parts using thermoplastic polymers [4,5,6]. FFF techniques have been employed in the fabrication of a variety of products, such as components for the aerospace and automotive industries, as well as devices and systems for pharmaceutical and biomedical applications [7,8]. Research on FFF has been expanding and diversifying in recent years, focusing on enhancing the performance and functionality of fabricated polymer components. The increasing variety of filament materials with diverse physical, mechanical, and electronic properties has advanced Multi-Material Additive Manufacturing (MMAM), with FFF enabling the creation of 3D printed parts featuring tunable properties through the combination of two or more materials with distinct characteristics [9,10]. Multi-material FFF parts can be created by combining two or more polymeric materials through extrusion, either using a single mixing nozzle or a multi-nozzle system, to produce a solid 3D printed structure [9,11,12,13]. Research groups have worked on the challenges of multi-material FFF techniques, including material compatibility [14], interfacial bonding strength [15], thermal processing requirements [16], customised printing materials [17], design and slicing complexity [18], and achieving mechanical property gradients [19]. However, no studies have investigated filament recognition to fully automate the multi-material printing process.

Research has been conducted to design FFF systems with sensors and machine learning models to enhance precision and reliability, ensuring that products meet the specified requirements while maintaining consistent print quality with minimal human intervention throughout the printing process. The adaptive combination of sensors and ML models has been widely employed to optimise AM process parameters and in situ process monitoring [20,21]. The printer process parameters, layer thickness, air gap, raster angle, build orientation, and road width, have been optimised for improving the material viscoelastic performance [22], minimising support material [23], estimating the dynamic modulus of elasticity [24], and improving wear resistance [25] and compressive strength [26]. In situ monitoring has been applied to increase the product quality and printing efficiency [20,27].

Although multi-spectral sensors and ML methods have been used for plastic recognition [28,29], this work represents the first implementation for filament recognition in additive manufacturing. In the context of the smart manufacturing paradigm, the future of AM is expected to operate fully autonomously. For this vision to become a reality, advancements in multi-material printing will need to focus on identifying materials and automatically adjusting the printer parameters such as bed and nozzle temperatures, to ensure high-quality prints. The primary challenge in multi-material FFF lies in optimising the printing process for specific materials. With a wide range of polymers available such as polylactic acid (PLA), acrylonitrile butadiene styrene (ABS), thermoplastic polyurethane (TPU), and thermoplastic copolyester (TPC), manual identification and adjustment of printer parameters can be both time-consuming and error-prone. This work aims to eliminate the manual marking of raw materials in FFF technology for additive manufacturing reducing the potential of human errors, by introducing a method that enables automated filament recognition and parameter adjustment. Furthermore, integrating this approach with an automatic filament changer allows for dynamic and autonomous filament loading, significantly enhancing the flexibility and efficiency of the printing process. Since each polymer type requires specific temperature and extrusion settings to ensure optimal printing quality and prevent defects, accurate material recognition is a crucial step in multi-material FFF. This growing diversity of printing materials highlights the increasing need for machine learning to automate the recognition and handling of different printing materials. By addressing this need, autonomous multi-material FFF systems can significantly enhance efficiency, reliability, and overall print quality.

This paper proposes a novel approach that leverages a multi-spectral sensor and machine learning methods to detect and identify polymer materials commonly used in additive manufacturing. The sensor collects spectral data across a range of wavelengths from filaments including polylactic acid (PLA), acrylonitrile butadiene styrene (ABS), ABS blended with carbon fibre, carbon fibre, thermoplastic copolyester (TPC), and thermoplastic polyurethane (TPU). The collected data are analysed using a comparative approach with a set of machine learning methods, including k-Nearest Neighbours (kNN), Logistic Regression, Support Vector Machine (SVM) and Multi Layer Perceptron (MLP), to evaluate their performance achieving accurate recognition of the printing material. The results from this study show that the combination of a spectral sensor and machine learning models can effectively identify filament types. Furthermore, this identification can contribute to the automatic adjustment of printer parameters to match the specific properties of the detected polymer, thereby enabling an autonomous printing process.

The rest of this paper is organised as follows: The low-cost sensor, filaments data, and recognition methods are presented in Section 2. The experiments and results are described in Section 3. The discussion and conclusion of this work are shown in Section 4 and Section 5, respectively.

## 2. Methods

### 2.1. Multi-Spectral Sensor

**Figure 1:**
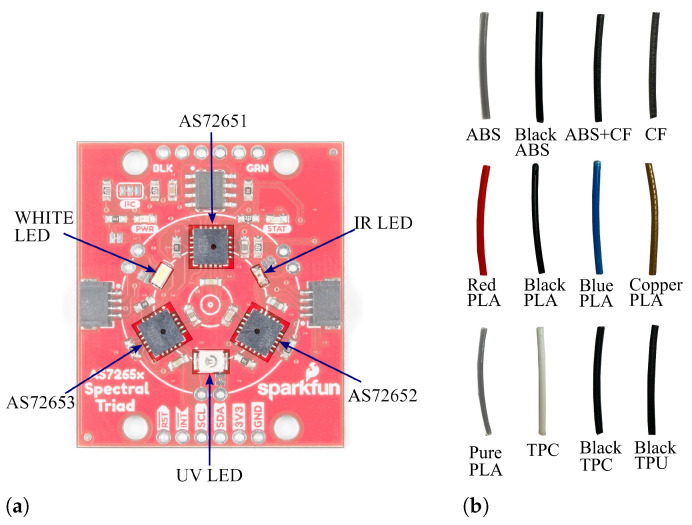
Low-cost spectroscopy sensor and filament samples. (**a**) Triad Spectral Sensor module from SparkFun Electronics [31]. (**b**) Examples of filaments used for data collection and recognition processes.

The multi-spectral chipset AS7265x, from asm OSRAM, Graz, Austria, is employed for the recognition of various filaments. The chipset includes three sensor devices, the AS72651, AS72652, and AS72653, capable of performing spectral identification across the visible to near-infrared (NIR) range (from 410 nm (ultraviolet, UV) to 940 nm (infrared, IR)). Each sensor device includes 6 optical filters capable of detecting light intensity across different spectral ranges. The AS72651, when combined with the AS72652 (spectral response range: 560 nm to 940 nm) and the AS72653 (spectral response range: 410 nm to 535 nm), constitutes the AS7265x 18-channel multi-spectral sensor chipset [30]. The Triad Spectroscopy Sensor, by SparkFun Electronics, combines the three multi-spectral sensors mentioned above in a single package, along with visible, UV, and IR LED light sources (see Figure 1a). This spectroscopy sensor is used in this work to design the filament recognition module.

### 2.2. Filaments Used for Data Collection

In the FFF printing method, a wide range of polymer materials is commonly used, spanning from simple to complex formulations. For the filament recognition experiment, we focussed on testing the method with a diverse selection of filaments. This included filaments of the same material in different colours, as well as those with the same colour but made from different materials. The filaments used in this study included PLA in red, blue, black, copper, and pure (transparent, without colour additives); transparent ABS; black ABS; ABS mixed with carbon fibre; carbon fibre; black and white TPC; and black TPU (Figure 1b). This variety of filament samples was chosen to thoroughly challenge the system’s ability to detect and identify the correct printing material accurately.

### 2.3. Compact Filament Recognition Module and Data Collection

**Figure 2:**
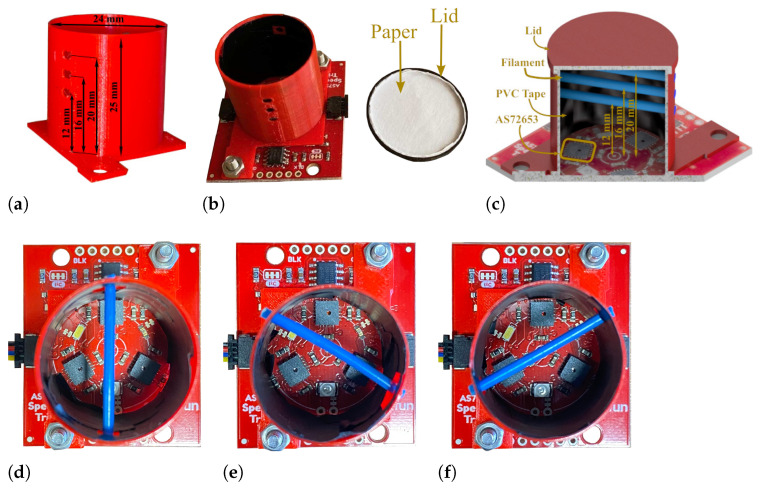
Shroud design for systematic data collection. (**a**) Shroud with three pairs of holes at heights of 12 mm, 16 mm, and 20 mm to place filaments for data collection. (**b**) The shroud is mounted on the board and covered with a lid. (**c**) Example of filaments placed at different heights for data collection. (**d**) Procedure for data collection from filaments using the AS72651 sensor, (**e**) the AS72652, and (**f**) the AS72651 sensor.

The filament detection design needs to be compact and small enough to be compatible with any 3D printer. To achieve this, we designed a shroud with a diameter of 24 mm and a height of 25 mm, printed using PLA material (Figure 2a). The shroud covers the sensors and light sources, ensuring that the emitted light remains within a controlled environment for accurate spectral detection. In addition, a lid was designed to securely cover the shroud, preventing interference from external environmental light, which could degrade the accuracy of the sensor measurement. Three shrouds were designed to position the filaments on each sensor during the data collection procedure and evaluate the responses of the sensors. Each shroud features three pairs of holes at heights of 12 mm, 16 mm, and 20 mm, enabling the filament to be positioned at different levels inside the shroud, while also simulating the filament’s passage during printing and facilitating data collection for printer control. To avoid interference from the PLA material of the shroud and lid on the measured filament signals, the interior of each shroud was covered with tape made of polyvinyl chloride (PVC) material, and a layer of paper was added to the inner side of the lid (Figure 2b). This setup ensures accurate sensor readings and data collection by reducing any potential effects of the PLA material used for the fabrication of the shroud and lid parts.

### 2.4. Data Collection Process

**Figure 3:**
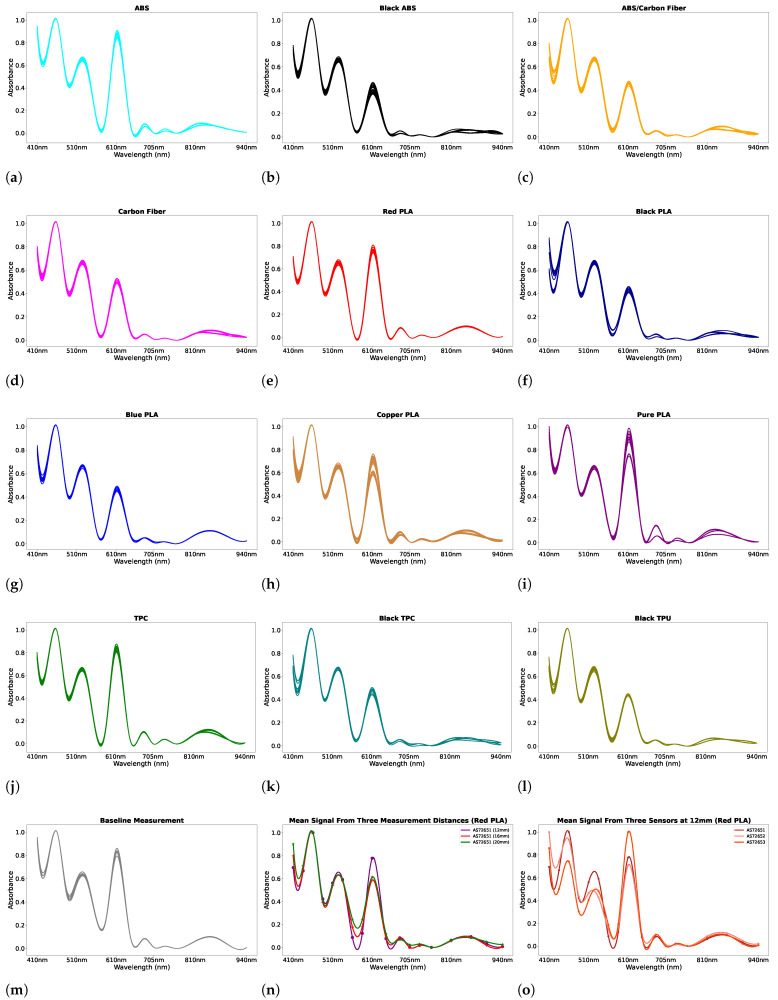
(**a**–**l**) Spectral information for each filament obtained from the multi-spectral sensor; filaments are positioned on the AS72651 sensor at a height of 12 mm. (**m**) Spectral information of baseline measurement. (**n**) The mean spectrum of Red PLA obtained at three distances on the AS72651 sensor. (**o**) The mean spectrum of the Red PLA filament collected at a 12 mm measurement distance using three different sensors.

An example of the data collection procedure is shown in Figure 2c where the blue filament is placed on the unit AS72653 with three different measurement distances. The data are collected from the filaments described in Section 2.2. During data collection, all sensors operated under UV, IR, and visible light conditions. A baseline measurement was recorded without any filaments to establish a reference dataset. The data collection process then began positioning the filaments on the AS72651 sensor (Figure 2d) at a measurement distance of 12 mm. Each filament was placed inside the shroud with a repetition of three times at the 12 mm measurement distance. This data collection approach was necessary as slight variations in filament alignment were observed to cause subtle changes in the signal. For each filament placement, 110 measurements were recorded per minute, resulting in a total of 330 measurements per filament for each distance. The same data collection process was repeated at measurement distances of 16 mm and 20 mm. This process was repeated placing the filaments on the other sensor units (AS72652 (Figure 2e) and AS72653 (Figure 2f)), ensuring consistency across all measurements. The combination of 3 different measurement distance positions resulted in 9 distinct configurations, allowing for a comprehensive evaluation to determine the optimal setup to achieve the best recognition performance. Figure 3a–m show the example of raw data measurements of the filaments and the baseline measurement from the 330 samples recorded from AS72651, AS72652, and AS72653 sensors when the filaments are placed on AS72651 sensor at a measurement distance of 12 mm. The *x*-axis represents the measured wavelengths, while the *y*-axis shows the normalised signal response. Figure 3n illustrates the mean spectrum of Red PLA collected at three different measurement distances using the AS72651 sensor. The plot indicates that the measurement distance influences the absorbance changes at certain wavelengths. Furthermore, Figure 3o shows the mean spectrum of the Red PLA filament collected at 12 mm measurement distance across three different sensors. This highlights that each sensor produces a distinct signal for the same filament, even when measured under identical conditions.

### 2.5. Machine Learning Procedure for Filament Recognition

**Figure 4:**
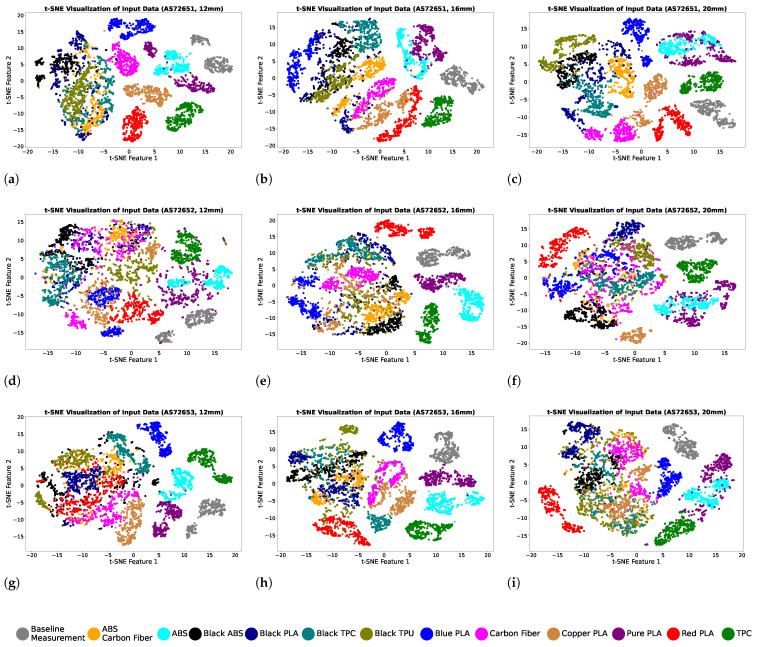
(**a**–**i**) t-SNE visualisation of the collected data from each data collection configuration.

The dataset for each configuration consists of 4290 readings, encompassing measurements from 12 different filaments and a baseline, resulting in an overall dataset size of 4290 × 18. During data collection, changes in filament orientation during positioning for the second and third rounds were observed to introduce slight variations in the signal. To replicate this effect, noise was added to the data using the standard deviation of signal changes for each filament. This modified dataset for each configuration is visualised in a 2D plane using the t-distributed stochastic neighbour embedding (t-SNE) technique to better understand the distribution of each filament data point [32]. This method reduces high-dimensional data into two dimensions, preserving the inherent structure and relationships between data points for better interpretability. The t-SNE technique was applied to the combined dataset from all configurations, with a perplexity parameter of 30 and 350 iterations. Figure 4 shows the cluster of filament data for all the data collection configurations. The dataset generated from AS72651 exhibits a diverse distribution after applying the t-SNE method, with distinct clusters forming for most filament types. In contrast, the datasets from AS72652 and AS72653 show more compact clustering, but with larger overlap among certain filament types. Overall, the visualisations demonstrate that the baseline and TPC filaments have distinct features and are easily distinguishable, forming clearly defined clusters. However, Black ABS, Black PLA, Black TPU, and ABS + carbon fibre exhibit similar feature sets, making them more challenging to distinguish. Among all configurations, the data distribution shown in Figure 4c collected at 20 mm measurement distance on the AS72651 sensor achieves the best separation and clustering of filament types.

**Figure 5:**
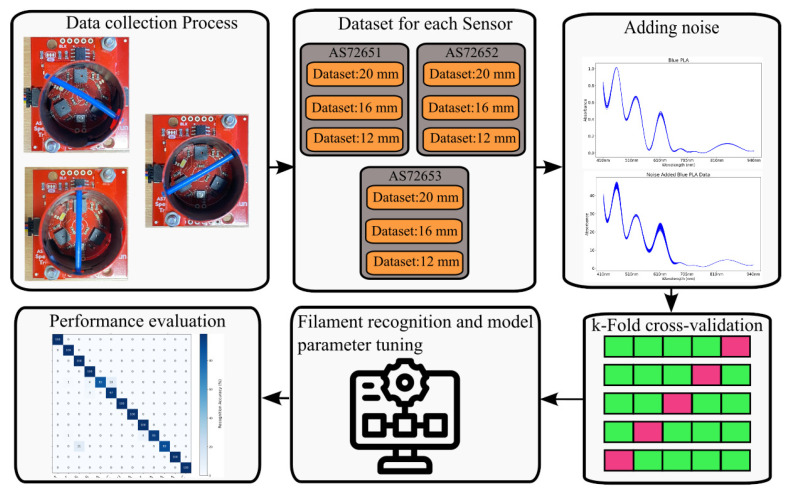
Overview of the data collection process and machine learning implementation for filament recognition.

Figure 5 shows the diagram of data collection and the implementation of machine learning methods for filament recognition. The classification of filaments is performed using k-Nearest Neighbours (kNN), Logistic Regression, Support Vector Machine (SVM), and Multi-Layer Perceptron (MLP), chosen based on material classification studies in the literature [28,29]. The input dataset size is 42,900 × 18, and the output size is 13 × 1 for each configuration. The dataset is divided into training and test subsets to assess the machine learning model’s performance. While 80% of the data was assigned to the training set, 20% was reserved for the test set. K-fold cross-validation, a common model validation method, is applied on the dataset, which splits the dataset into K equal folds, using each fold once as a test set while training on the rest. This process is repeated K times, and the results are averaged for a reliable performance estimate. Here, 5-fold cross-validation is utilised to assess our model’s robustness and generalisation. A common challenge in building ML models is to identify the optimal hyperparameters, as arbitrarily selecting and testing them can be time-consuming and computationally expensive. In this work, hyperparameter tuning is performed systematically using the grid search method. The process involves defining hyperparameter ranges, training and evaluating the model for each combination using cross-validation, and selecting the set that maximises performance based on a predefined metric. A 4-fold training and 1-fold validation approach is applied for all ML models, with tuning tailored to each algorithm.

For the kNN, the tuned parameters include the number of neighbours, weights, and distance metric. In most of the optimised kNN models, the best performing parameters have been obtained to be 5 neighbours, distance-based weighting, and Manhattan distance metric. For Logistic Regression, the tuning process focusses on the regularisation type (penalty), regularisation strength (C), solver, and the maximum number of iterations. In most of the optimised LR models, the regularisation type was L1, the regularisation strength (C) ranged between 8, 9, and 10, the solver was set to liblinear, and the maximum number of iterations was 3000. For SVM, the regularisation parameter (C), kernel type, and kernel coefficient (gamma) are optimised. In most of the optimised SVM models, the best-performing parameters have been obtained to be a regularisation parameter (C) of 10, a kernel coefficient (gamma) of 0.1, and an Radial Basis Function (RBF) kernel type. For MLP, the tuning process involves the number of epochs, batch size, and architecture, including the number of layers and neurons. In most of the optimised MLP models, the architecture consisted of 3 hidden layers with 18, 32, and 18 neurons, a batch size of 8, and 100 epochs. The activation function for the hidden layers was Rectified Linear Unit (ReLU), while the output layer used a softmax activation function. Additionally, the Adaptive Moment Estimation (Adam) optimisation algorithm was employed to update the network parameters during the training phase.

The performance of the computational methods used in the recognition process is assessed through precision, recall, F1-score, and accuracy, which are widely utilised metrics for evaluating the performance of ML methods [33,34,35]. These metrics are calculated based on information from the following: correctly recognising the target class (true positive, *TP*), correctly recognising the non-target class (true negative, *TN*), incorrectly recognising the target class (false positive, *FP*), and incorrectly recognising the non-target class (false negative, *FN*). The formulas for the metrics are as follows:

(1) $$P=\frac{TP}{TP+FP}\times 100\%$$

(2) $$R=\frac{TP}{TP+FN}\times 100\%$$

(3) $$F1=\frac{2PR}{P+R}=\frac{2TP}{2TP+FP+FN}\times 100\%$$

(4) $$Accuracy=\frac{TP+TN}{TP+TN+FP+FN}\times 100\%$$

where *P*, *R*, and F1 represent the metrics for precision, recall, and F1-score, respectively.

## 3. Experiments and Results

**Figure 6:**
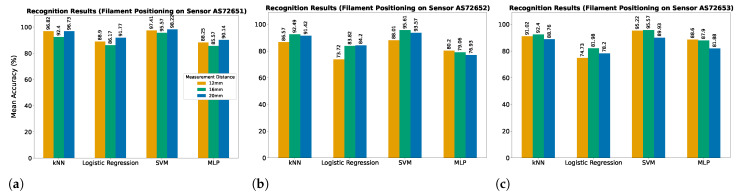
The average recognition accuracy of the machine learning models obtained through a 5-fold cross-validation approach; data collected positioning the filaments on the sensors: (**a**) AS72651, (**b**) AS72652, and (**c**) AS72653.

This section presents the results of filament recognition using the spectroscopy module and kNN, Logistic Regression, SVM, and MLP methods from three different measurement distance and position configurations. Figure 6 shows the mean recognition accuracy obtained from a 5-fold cross-validation approach. Among the three tested data measurement positions (on AS72651, AS72652, and AS72653 sensor units), the AS72651 unit consistently achieved the highest accuracy across all distances with the Support Vector Machine (SVM) model performing best, reaching 98.22% accuracy at 20 mm with a response time ≈2 ms. The k-Nearest Neighbours (kNN) model demonstrated good performance, with accuracies around 96%. In contrast, Logistic Regression and Multi-Layer Perceptron (MLP) exhibited slightly lower accuracy reaching around 88% accuracy. For the case of positioning the filament on the AS72652 sensor unit, the overall accuracy was lower, with SVM again achieving the highest accuracy of 96% at 16 mm. The kNN model followed closely at 92%, while Logistic Regression and MLP obtained reduced accuracies, ranging from 73% to 80%. The highest recognition performance was obtained on the AS72653 sensor unit at a measurement distance of 16 mm where SVM and kNN achieved accuracies of 95.57% and 92.40%, respectively. MLP and Logistic Regression reached their maximum accuracies of 88.6% at 12 mm measurement distance and 81.98% at 16 mm measurement distance, respectively.

**Figure 7:**
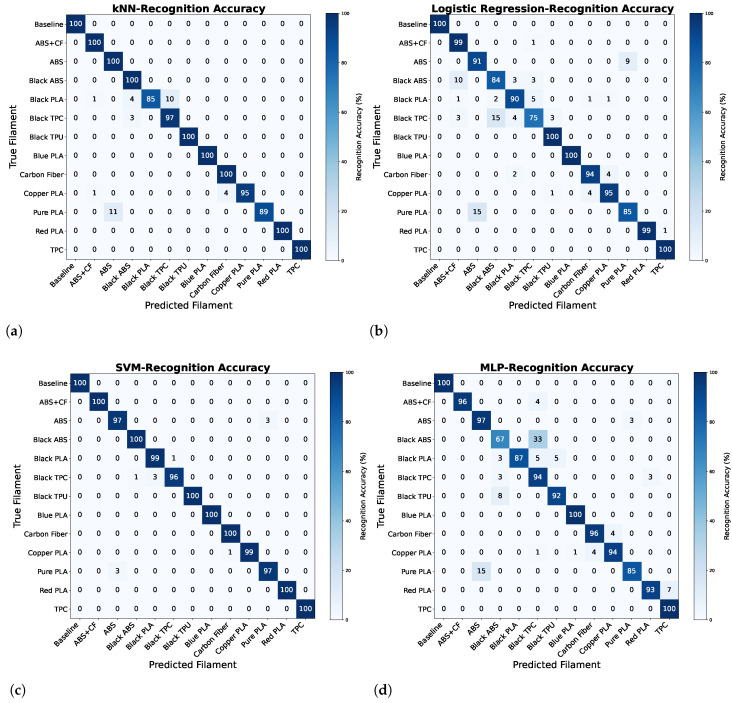
The highest recognition results achieved using data collected at a 20 mm measurement distance on the AS72651 sensor: (**a**) k-Nearest Neighbours (kNN), (**b**) Logistic Regression, (**c**) Support Vector Machine (SVM), and (**d**) Multi-Layer Perceptron (MLP).

The confusion matrices in Figure 7 show the best filament recognition results of kNN, Logistic Regression, SVM, and MLP models from the configuration AS72651 at 20 mm distance. SVM achieved over 98.95% accuracy for all filament types, making it the most reliable model for the recognition process. The kNN model performed well with the accuracy exceeding 96% for most classes. However, minor misclassifications were observed, particularly between similar filaments such as Black PLA and Black TPC, and ABS and Pure PLA. Logistic Regression, while effective for certain classes such as Baseline and TPC (100% accuracy), struggled with other filaments such as Black TPC (75% accuracy) and Black ABS (84% accuracy), showing a higher degree of confusion. The Multi-Layer Perceptron (MLP) model exhibited variable performance with high accuracy for certain filaments, e.g., TPC and Red PLA (100%), but struggled significantly with Black ABS (67%) and Pure PLA (85%). Overall, the results highlight the superiority of the SVM model for filament classification using spectroscopy data, particularly for the case of distinguishing similar filaments. Furthermore, Table 1 summarises the performance of kNN, Logistic Regression, SVM, and MLP for the recognition of filaments, evaluated using standard metrics: accuracy, precision, recall, and F1-score.

## 4. Discussion

This research introduces a method for recognition of filaments commonly used in the FFF printing process by employing a multi-spectral Triad Spectroscopy Sensor module and a set of machine learning algorithms. The study focussed on the classification of PLA, ABS, TPU, TPC, carbon fibre, and ABS/carbon fibre filaments. A compact module with an integrated shroud was specifically designed and mounted onto the Triad sensor module for systematic data collection. The designed module is low-cost (70 GBP), and can be easily used by other researchers. Three shrouds were developed to collect spectral data by positioning the filaments at different measurement distances on different sensors. The collected spectral signals consisted of 18 wavelengths ranging from 410 nm to 940 nm. This approach was designed to analyse the impact of various measurement configurations on the performance of filament type recognition.

The data distribution from different filaments was analysed using the t-SNE method to visualise the data and evaluate the similarity of the features between different filaments. The t-SNE plots revealed that the data collected on the AS72651 sensor formed better-defined clusters, indicating that this sensor is more effective at capturing filament-specific features. Subsequently, the data from each configuration were used in four machine learning models: kNN, Logistic Regression, SVM, and MLP. A grid search method was applied to each model to optimise the hyperparameters and enhance the classification performance. The results showed that the data collected by positioning the filaments on the AS72651 sensor outperformed other filament positions with respect to classification accuracy across all machine learning models, consistent with the t-SNE analysis, which indicated a better clustering of data. Furthermore, the classification results highlighted the impact of the measurement distance on model performance, with variations in signals affecting the accuracy. Among the evaluated models, SVM consistently delivered the best performance across all configurations, achieving the highest mean accuracy of 98% at a 20 mm measurement distance on the AS72651 sensor. These recognition results show that the UV light frequency absorbed by the AS72651 sensor plays a critical role in shaping the sensor sample for the recognition process. The confusion matrices for the models with the highest recognition performance revealed that black-coloured PLA, TPU, TPC, and ABS exhibit similar absorbance patterns, leading to some classification challenges. Similarly, ABS and Pure PLA, being transparent materials, showed an overlapping absorbance at similar wavelengths. Blue-coloured PLA and TPC, which have different colours compared to other samples in the dataset, were recognised with 100% accuracy. These results indicate that colourants in the filaments affect recognition performance due to the similarity in sensor signals after light absorption. Based on this analysis, we can expect that changes in the colour of the filament due to surface aging or colour inconsistencies may introduce prediction errors. The results also suggest that filament colour is not the sole factor influencing the sensor signal. Notably, PLA and TPU filaments of the same colour produced different signals, highlighting the impact of material composition on the recognition process. Another aspect that might affect the performance of the prediction accuracy is the diameter of the filament, which was not investigated in this work and it is an interesting aspect, together with surface aging, for the future work.

This research has shown that filament type for additive manufacturing with fused filament fabrication can be predicted accurately using spectroscopy data and machine learning. This work still has some limitations that can be investigated in the future work. For instance, black-coloured PLA with various additives can be challenging to identify accurately. Therefore, the measurement system may require more in-depth information about the material. Incorporating sensors that provide additional data would enhance the flexibility of the approach, allowing the system to better handle the recognition of untrained materials and adjust the printer settings accordingly. Additionally, the current system keeps the filament static in the shroud while data are collected for the prediction process. However, integrating the shroud in the real-time additive manufacturing process with the filament passing continuously through the shroud might affect the quality of the data collected, adding significant noise and potentially impacting on the accuracy of the prediction output. These limitations are interesting aspects for the future analysis and real-time implementation of the prediction module within an additive manufacturing process.

The findings of this work highlight that model selection, measurement distance, and position are critical factors that influence recognition performance. These findings emphasise the need for sensor and parameter optimisation in filament classification tasks. The research demonstrates the ability to accurately identify various types and colours of filaments that can contribute to the design of enhanced and autonomous multi-material printing processes. The promising results suggest potential applications for dynamically modifying printer parameters to enhance print quality and efficiency.

## 5. Conclusions

This study successfully demonstrated a method for recognition of commonly used filaments (PLA, TPU, TPC, carbon fibre, ABS, ABS/carbon fibre) in the FFF printing process by integrating a multi-spectral Triad Spectroscopy Sensor module with machine learning algorithms (kNN, Logistic Regression, SVM, and MLP). Using three different sensors (AS72651, AS72652, and AS72653) from the module and collecting spectral data at varying measurement distances, this study comprehensively analysed the effect of measurement configurations on filament recognition performance. Positioning the filaments on the AS72651 sensor consistently outperformed the other filament positions, with the SVM model achieving the highest accuracy of 98% at a measurement distance of 20 mm. Overall, this research presents a compact and low-cost filament recognition module capable of accurately identifying a wide range of filament types and colours. The proposed method shows great potential for enhancing the autonomy of multi-material printing processes and versatility in additive manufacturing applications.

## Figures and Tables

**Table 1:** Performance metrics (accuracy, precision, recall, and F1-Score) of the best-performing classifiers for filament recognition.

Best Classifier	Accuracy	Precision	Recall	F1-Score
kNN	97.20%	97.39%	97.20%	97.16%
Logistic Regression	93.12%	93.10%	93.12%	93.04%
SVM	98.95%	98.95%	98.95%	98.94%
MLP	92.65%	93.30%	92.65%	92.69%

## Data Availability

Data created during this research work is openly available from the University of Bath Research Data Archive at https://doi.org/10.15125/BATH-01501.
